# Synthesis of [B,Al]-EWT-Type Zeolite and Its Catalytic Properties

**DOI:** 10.3390/molecules27175625

**Published:** 2022-08-31

**Authors:** Youju Wang, Yongyue Bai, Pohua Chen, Qiang Chen, Yongrui Wang, Xingtian Shu

**Affiliations:** 1 State Key Laboratory of Catalytic Materials and Reaction Engineering, Research Institute of Petroleum Processing, SINOPEC, Beijing 100083, China; 2 Beijing National Laboratory for Molecular Sciences, College of Chemistry and Molecular Engineering, Peking University, Beijing 100871, China; 3 School of Chemical Engineering and Technology, Sun Yat-sen University (Zhuhai Campus), Zhuhai 519082, China

**Keywords:** EWT, synthesis, physico-chemical property, ketalization, catalytic reaction

## Abstract

EWT zeolite belongs to ultra-large pore zeolite with the 10MR and 21MR channels, which has good thermal stability, certain acid strength and good application prospects in petroleum refining and petrochemical reactions. However, EWT zeolite has fewer medium/strong acid sites, especially Brönsted acid sites, which makes it difficult to apply to acid-catalyzed reactions. The regulation of acid amount and distribution was achieved by boron and aluminum substitution into the siliceous framework of EWT. The physico-chemical properties of the samples were characterized by XRD, SEM, N_2_ adsorption-desorption, XRF, ICP, Py-IR, NH_3_-TPD and ^11^B & ^27^Al & ^29^Si MAS NMR. The results show that quantities of boron and aluminum elements can occupy the framework of [B,Al]-EWT to increase the density of medium and strong acid centers, with more acidity and Brönsted acid centers than EWT zeolite. In the reaction of glycerol with cyclohexanone, the conversion of the sample (U-90-08-10/U-90-H-HCl) is significantly higher than that of the EWT sample, approaching or exceeding the Beta zeolite. A catalytic activity study revealed a direct correlation between the Brönsted acidic site concentration and the activity of the catalyst. The U-90-08-10-H catalyst was also considerably stable in the catalytic process. This work shows, for the first time, that extra-large pore zeolites can be used in industrial acid-catalytic conversion processes with excellent catalytic performance.

## 1. Introduction

Zeolites have found widespread uses as catalysts in petroleum refining and petro-chemistry, and as selective adsorbents in separation and purification. Over the last several decades, a large number of zeolite structures have been synthesized through the use of organic additives such as amines and alkylammonium ions. The number of new zeolite structures is 255 (see the International Zeolite Association (IZA) website at http://www.iza-structure.org, accessed on 1 August 2022). Zeolites are porous crystalline materials composed of SiO_4_ and AlO_4_ tetrahedra. These basic building blocks interconnect to form networks of channels and cavities of adequate size to adsorb molecules and ions.

Ultra-large pore zeolites break through the pore limitation of molecular sieves and show advantages in increasing the reactivity of macromolecules, improving product selectivity, prolonging the life of zeolites, and are expected to show application prospects in petrochemical production. EMM-23 is the first stable, three-dimensional extra-large pore zeolite, which was discovered by the ExxonMobil Research & Engineering Co. Inc. [1]. The framework topology of EMM-23 has been assigned the three-letter code EWT by the Structure Commission of IZA. Its pore system consists of trilobe-shaped pores that are bound by 21–24 tetrahedral atoms with 10.8 × 4.6 Å [2]. These extra-large pores are intersected perpendicularly by a two dimensional 10-ring channel system with 5.1 × 5.1 Å. EMM-23 was discovered from preparations in gels with Si/Al > 100 using either 1,1-(pentane-1,5-diyl)bis(1-propylpyrrolidinium) hydroxide (PPPH) or 1,1-(hexane-1,6-diyl)bis(1-propylpyrrolidinium) hydroxide (HPPH) as the OSDA. Xiao et al. proposed a method for EMM-23 using polyquaternium as a template agent [3]. Mu et al. also described the synthesis, characterization and catalytic property of RZM-3 and B-RZM-3 zeolites with EWT structure [4,5]. However, RZM-3 and B-RZM-3 zeolites have less amounts of medium/strong acids, especially Brönsted acid sites, and lower B/L ratio, which makes them difficult to apply to acid-catalyzed reactions.

Catalytically active zeolites can be obtained by substitution of trivalent metals, such as Al, for Si in the siliceous framework. Each substitution creates a negative charge on the lattice, which is compensated by a proton or cation. When the negative charge is compensated by a proton, an acidic bridging hydroxyl group (Brönsted acid site) is created. The acidity of zeolites is directly related to the amount and siting of A1 incorporated into the framework. Most commercial zeolite catalysts have framework Si/A1 ratios of 1–30 with medium strength Brönsted acid centers [6,7,8,9,10]. However, it is difficult to obtain RZM-3 with appropriate acid amount and distribution through modification of synthesis conditions and post-synthesis treatment.

Heteroatom incorporation is an effective method to adjust the acidity, owing to the competitive occupancy of various framework T sites between the heteroatom and aluminum [11,12]. For ZSM-5 zeolite, the boron incorporation could markedly improve the catalytic performances of zeolites in MTH through regulating the framework aluminum (Al_F_) siting and acid distribution, which brought about a distinct change in the chemical shift of 4-coordinated aluminum species in the ^27^Al MAS NMR spectra of ZSM-5 [13,14,15].

In the present contribution, the boron and aluminum entrance into the framework was carried out on [B,Al]-EWT zeolite by hydrothermal synthesis method. The crystal structure and structure-related properties of [B,Al]-EWT, including physico-chemical and catalytic performance in catalysis, were listed. The data provide persuasive evidence that the regulation of acid amount and distribution in [B,Al]-EWT was achieved through boron incorporation, and the relation between the catalytic performance of [B,Al]-EWT in ketalization of glycerol with cyclohexanone and its Brönsted acidity was clarified. The insights shown in this work should be of great benefit to the development of the ultra-large pore zeolites in acid-catalyzed reactions.

## 2. Result and Discussion

### 2.1. The Preparation and Characterization of [B,Al]-EWT by Direct Synthesis

**Figure 1:**
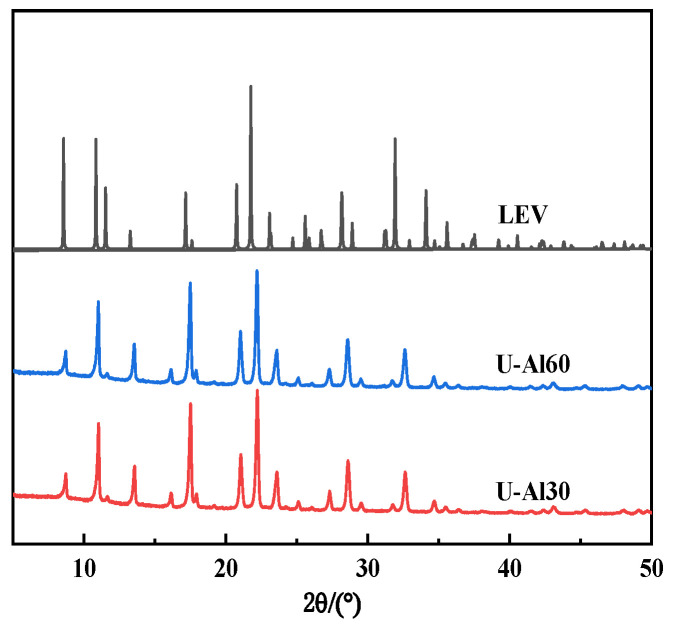
The XRD pattern of samples with *n*(SiO_2_)/*n*(Al_2_O_3_) = 30.

**Figure 2:**
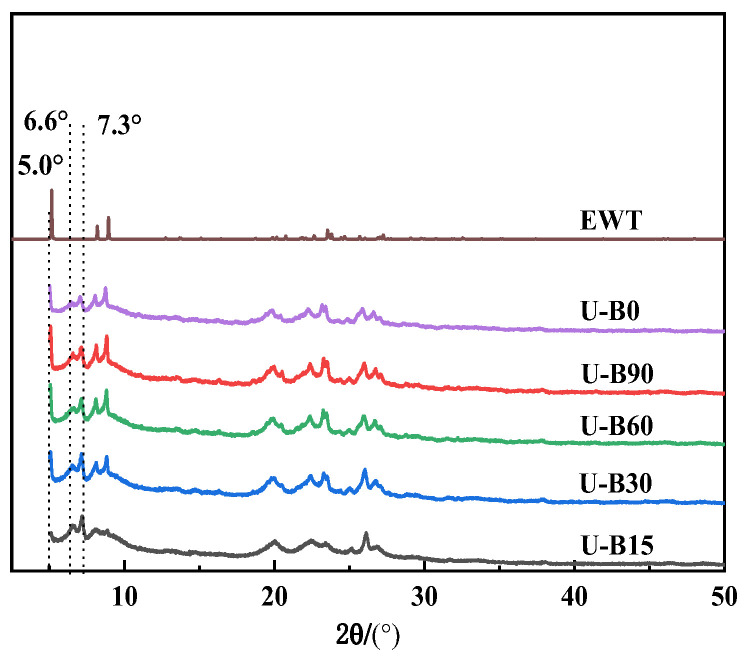
The XRD pattern of samples with different *n*(SiO_2_)/*n*(B_2_O_3_) ratios.

**Figure 3:**
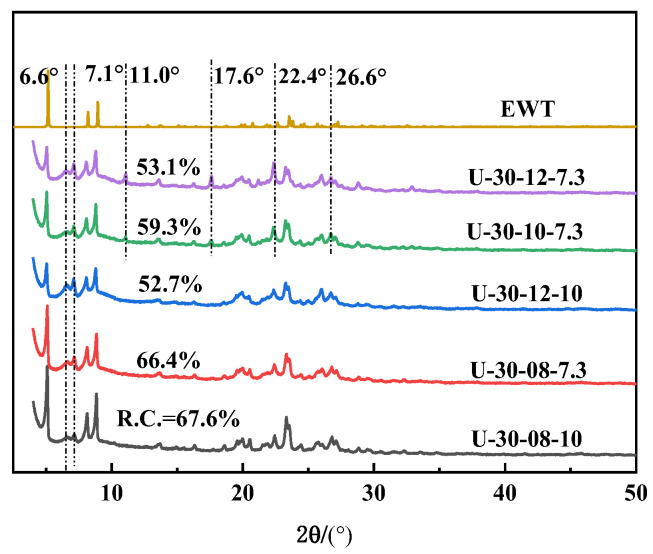
The XRD pattern of samples with *n*(SiO_2_)/*n*(B_2_O_3_) = 30.

**Figure 4:**
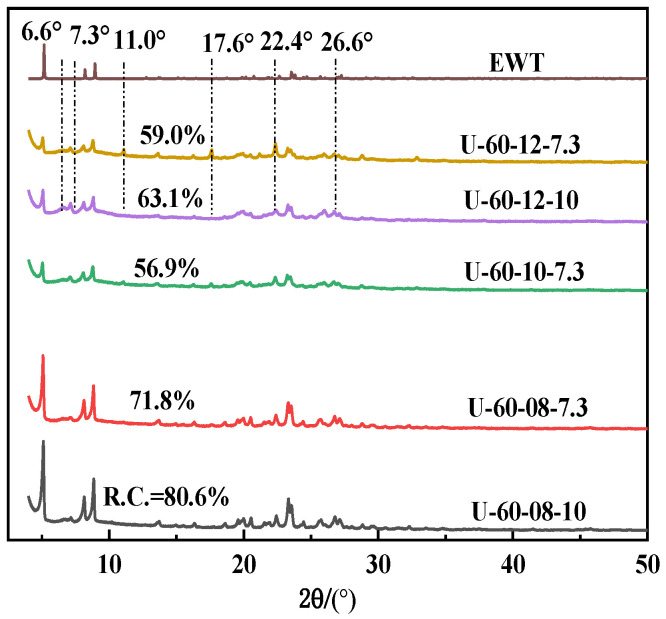
The XRD pattern of samples with *n*(SiO_2_)/*n*(B_2_O_3_) = 60.

**Figure 5:**
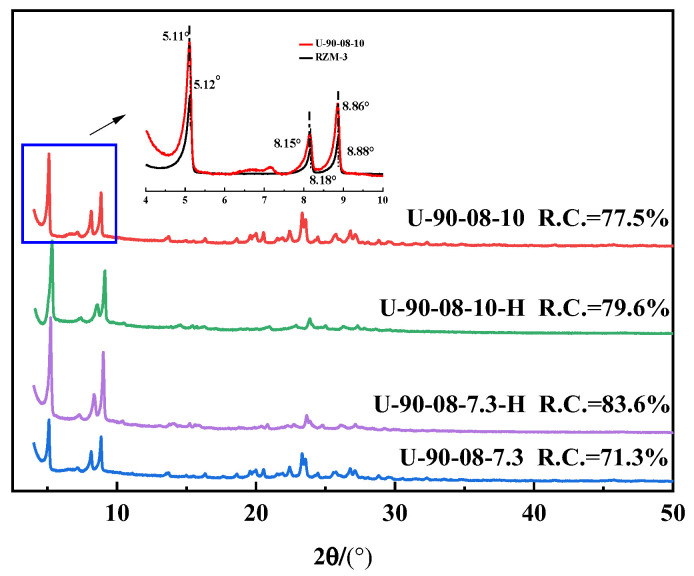
The XRD pattern of samples with *n*(SiO_2_)/*n*(B_2_O_3_) = 90.

To improve the acid content and acid strength of EWT, [B,Al]-EWT zeolites with varied SiO_2_/B_2_O_3_ and SiO_2_/Al_2_O_3_ ratio were synthesized (Table 1). First, the samples were synthesized under feed *n*(SiO_2_)/*n*(Al_2_O_3_) of 30 with different *n*(SiO_2_)/*n*(B_2_O_3_) (entry I). The results show that the product is LEV zeolite (Figure 1); Second, the feed n(SiO_2_)/n(Al_2_O_3_) was increased to 60 (entry II). The XRD patterns of the samples indicate that the characteristic peaks of MCM-22 and EWT appear in all samples at 2θ = 6.6/7.3° and 2θ = 5.0°, which show the products are miscible phases containing EWT and MCM-22 zeolites (Figure 2). The intensity of the characteristic peak (2θ = 5.0°) of the EWT gradually increased with the increase of *n*(SiO_2_)/*n*(B_2_O_3_) ratio, and the intensity of the characteristic peak of U-B90 was the highest; Third, the ratios of *n*(NaOH)/*n*(SiO_2_) and *n*(H_2_O)/*n*(SiO_2_) were modified under the condition of *n*(SiO_2_)/*n*(B_2_O_3_) = 30/60 to prepare [B,Al]-EWT with high crystallinity (entry III). From Figure 3, the samples U-30-12-10/U-30-10-7.3/U-30-12-7.3 with the *n*(NaOH)/*n*(SiO_2_) ratios of 0.10/0.12 appeared the characteristic peak at 2θ = 6.6/7.1°, 2θ = 11.0/17.6/22.4/26.6°and 2θ = 5.0°, which show that the products are miscible phases containing MCM-22, MEL and EWT zeolites; Reducing the *n*(NaOH)/*n*(SiO_2_) ratio to 0.08, samples U-30-08-10/7.3 exhibits a higher proportion of EWT, although it still contained a small amount of MCM-22 zeolite impurity. Similarly, the samples U-60-12-10/U-60-10-7.3/U-60-12-7.3 with the *n*(NaOH)/*n*(SiO_2_) ratios of 0.10/0.12 were miscible phases containing MCM-22 and EWT zeolites. Especially samples U-60-08-10/7.3 with lower *n*(NaOH)/*n*(SiO_2_) ratio of 0.08 were pure-phase EWT zeolite with higher crystallinity (80.6%/71.8%) (Figure 4); Fourth, [B,Al]-EWT with a *n*(SiO_2_)/*n*(B_2_O_3_) ratio of 90 were obtained under the condition of *n*(NaOH)/*n*(SiO_2_) = 0.08 (entry IV). The crystallinity of samples U-90-08-10/7.3 were 77.5%/71.3% (Figure 5).

**Figure 6:**
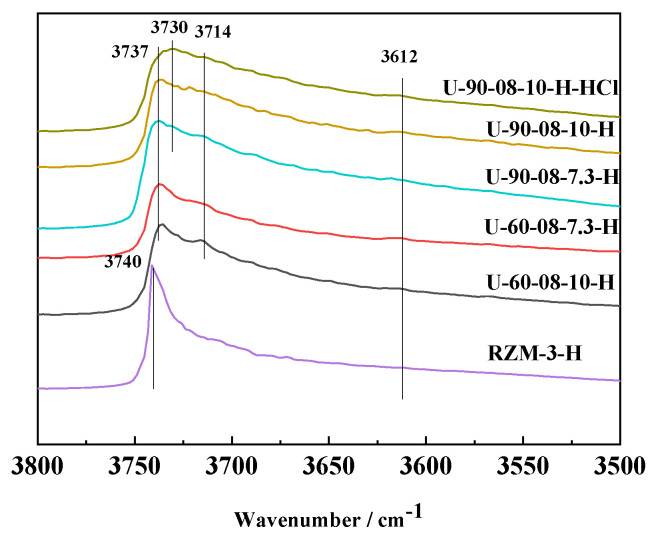
The OH-IR spectra of samples.

From Figure 5, the characteristic peaks of the U-90-08-10 sample are shifted to a lower angle compared with EWT zeolite, which may be caused by the change of the unit cell parameters due to element boron entering the zeolite framework (B-O bond length is 0.147 nm, and the Si-O bond length is about 0.161 nm [16]). The samples remained structurally stable after calcination (Figure 5). Also, whether the boron element was entered the zeolite framework can be seen from the OH-IR spectrum (Figure 6). The *v*(OH) region of the IR spectrum of [B,Al]-EWT-H contains bands at around 3612, 3714 and 3740 cm^−1^. The 3612 cm^−1^ has been assigned to an acidic bridging OH by Si-O-Al, while 3740 cm^−1^ is attributed to terminal SiOH or extra-framework silica gel. The spectra of EWT contain the 3740 cm^−1^ peak, but the [B,Al]-EWT samples (U-90-08-10/7.3) were shifted to 3737 cm^−1^, as seen in Figure 6. It has been attributed to the weak Brönsted site of B-O-Si, which is consistent with that of H[B]-ZSM-5 zeolite [11].

**Figure 7:**
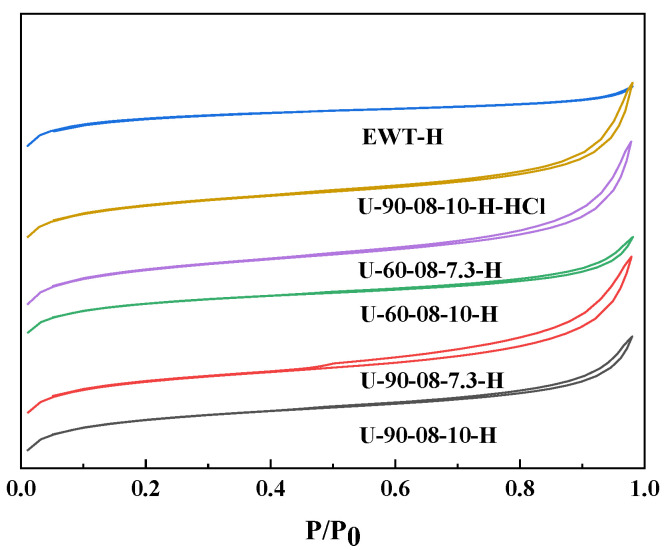
The isotherm plots of samples.

**Figure 8:**
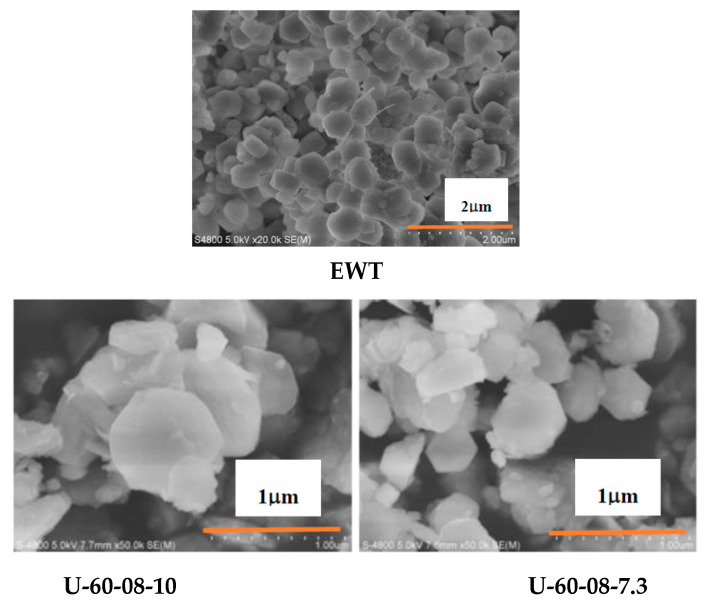

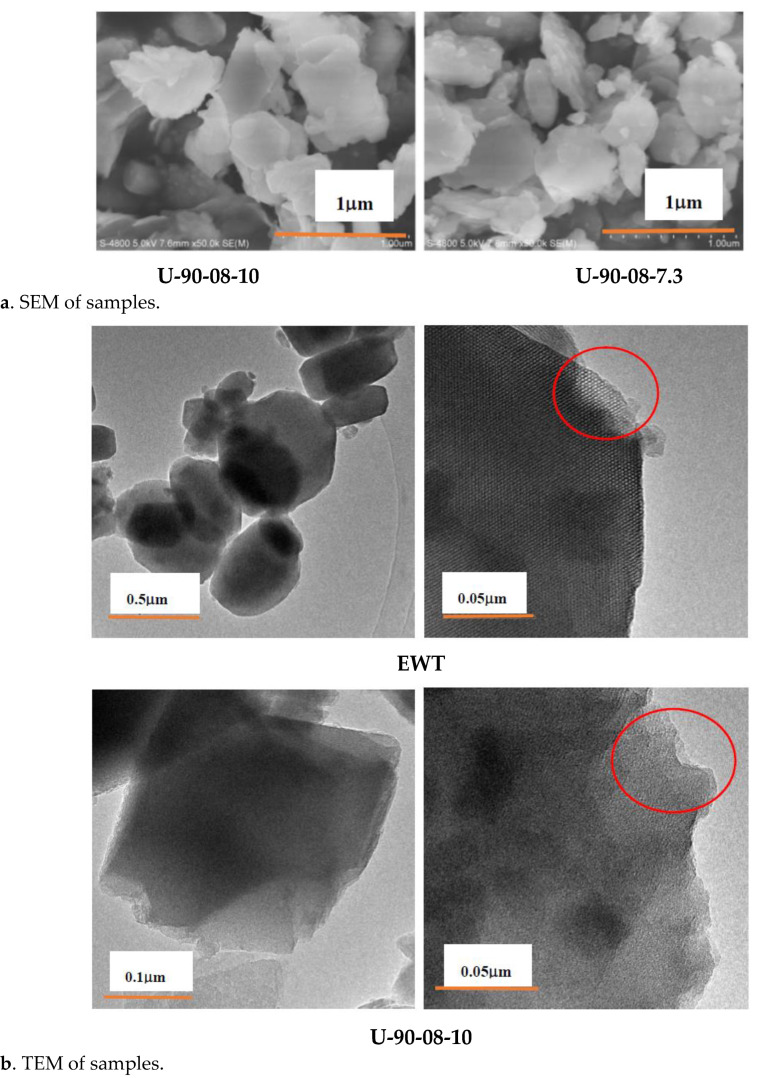
(**a**) SEM (**b**) and TEM images of samples.

Table 2 shows the elemental compositions and textural properties of samples. The Si/Al ratio of [B,Al]-EWT zeolites are lower than that of designated value (Ca.60) in the synthesis gels. The Si/B ratio of [B,Al]-EWT zeolites are higher than those of the synthesis gels, suggesting that the incorporation of less boron element into the EWT framework may improve the aluminum element incorporation into the zeolites. This is different from that of EWT zeolite whose Si/Al ratio is lower than the feeding ratio (Ca.120). The Al/B ratio are in the range of 3.0~4.4. The results of nitrogen physisorption on the H-form [B,Al]-EWT zeolites exhibit samples have lower surface area and larger pore volume compared to EWT (Figure 7), which were related to lower crystallinity of samples. The SEM and TEM images of samples are shown in Figure 8a,b, respectively. All samples are in the hexagonal shape of a typical EWT with particle size of around 0.4–0.8 μm, further illustrating that [B,Al]-EWT causes little disturbance to the textural properties of EWT crystals (as shown in the red circles of Figure 8b).

**Figure 9:**
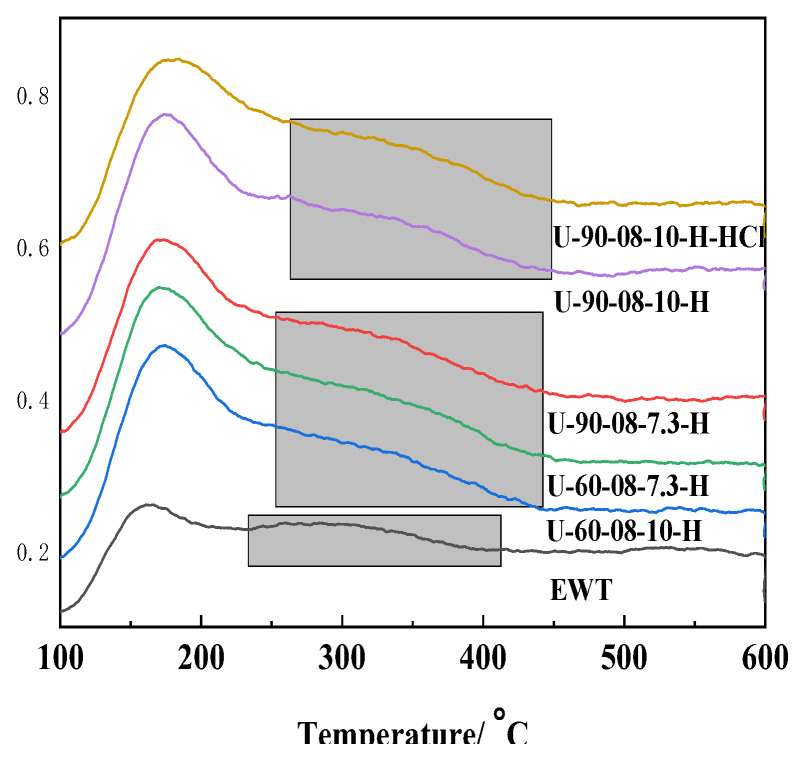
The NH_3_-TPD profiles of samples.

From Figure 9, all the H-form samples are quite similar in their NH_3_-TPD profiles: two desorption peaks appear at around 100–290 °C and 290–600 °C, which were assigned to weak and strong acid sites, respectively. The higher the acid strength of the zeolites, the higher the ammonia desorption temperature. Sample U-90-08-10 showed the highest desorption temperature among other samples. The quantitative NH_3_-TPD results given in Table 3 demonstrated that samples have more total acidity than EWT zeolite, and sample U-90-08-10 showed the highest amount of acidity among other samples.

**Figure 10:**
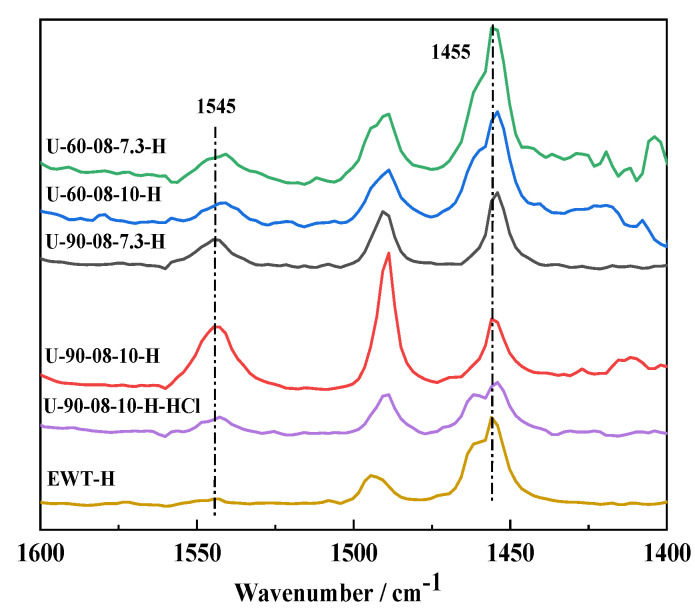
The Py-IR spectra of samples.

Unlike NH_3_-TPD, Py-IR is able to differentiate the Bronsted and Lewis acid sites. The bands associated with the pyridinium ion adsorbed at Brönsted acid sites and pyridine coordinated to Lewis acid sites appear at 1545 and 1455 cm^−1^, respectively, as shown in Figure 10. The quantitative Py-IR results given in Table 3 demonstrated that these samples have higher density of Brönsted acid sites and B/L ratio than EWT zeolite, and sample U-90-08-10 showed the highest amount of Brönsted acid sites and ratio of B/L among the samples.

**Figure 11:**
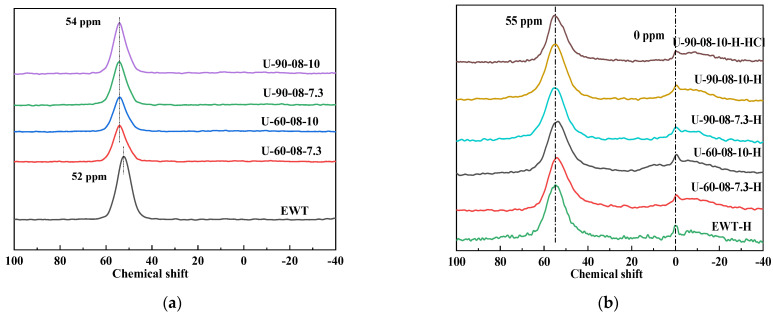
The ^27^Al MAS NMR spectra of (**a**) as made and (**b**) calcined samples.

As the Brönsted acid sites in zeolites are primarily derived from the framework aluminum (Al_F_) species [18], ^27^Al MAS NMR spectra were used to investigate the aluminum coordination. As displayed in Figure 11, the intense band around 52–55 ppm and weak peak at 0 ppm can be assigned to 4-coordinated framework (Al_F_) and 6-coordinated extra-framework aluminum (Al_EF_), respectively [19]. The chemical shift at 53 ppm (U-60-08-10/U-60-08-7.3/U-90-08-10/U-90-08-7.3) and 55 ppm (EWT) can be assigned to Al_F_ (Figure 11a). The difference of the chemical shift is probably due to the different distributions of Al over tetrahedral sites (T sites) with different acidic properties [13,14,15]. From Figure 11b, extra-framework aluminum species appeared at 0 ppm for the calcined samples, and the samples have different fraction of Al_F_ species, the fraction of Al_F_ species from U-90-08-10 and U-90-08-10-HCl are higher than others, which is in line with the observation determined by NH_3_-TPD and Py-IR(Table 3).

**Figure 12:**
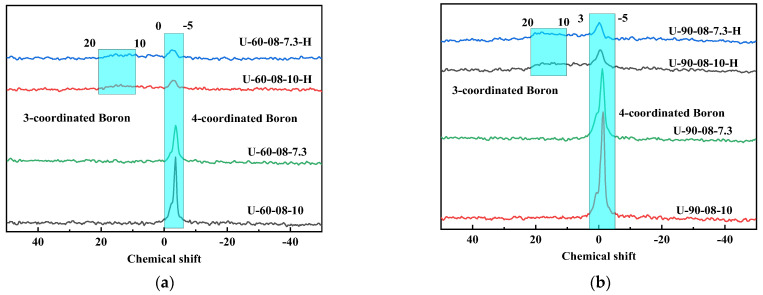
^11^B MAS NMR of (**a**) as made and (**b**) calcined samples.

^11^B MAS NMR spectra were used to investigate the boron coordination. As displayed in Figure 12, the intense band around −5–0 ppm and 10–20 ppm can be assigned to 4-coordinated framework boron (B(IV)) and 3-coordinated framework boron (B(III)), respectively [19]. Most of the boron in samples U-60-08-10/U-60-08-7.3/U-90-08-10/U-90-08-7.3 exist in the B(IV) form (Figure 12a). From Figure 12b, the B(III) species appeared at 10–20 ppm for the calcined samples, which may generate more Lewis acid centers and abundant nested hydroxyl groups. B(III) species are more easily removed by acid washing. Therefore, compared to U-90-08-10, sample U-90-08-10-H-HCl shows higher B/L ratio (Table 3 and Figure 10) and more hydroxyl groups at 3730 cm^−1^ (Figure 6).

**Figure 13:**
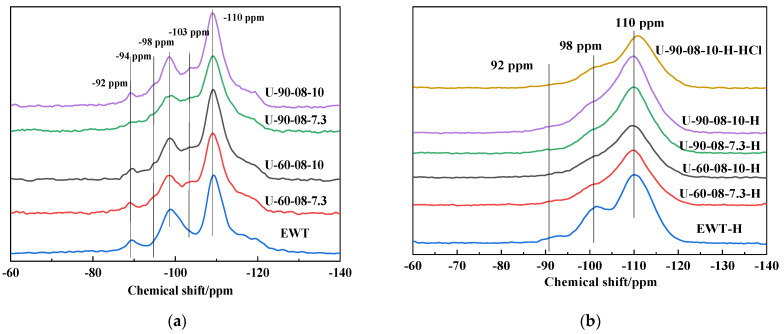
^29^Si MAS NMR of (**a**) as made and (**b**) calcined samples.

^29^Si MAS NMR spectra were used to investigate the silicon coordination. As displayed in Figure 13, the chemical shifts of −110/−98/−89 are assigned to the characteristic peak positions of Q^4^/Q^3^/Q^2^, respectively, and the chemical shifts of −103/−93 may be assigned to Si(1T)/Si(2T) connected ^29^Si atoms (T is boron or aluminum atom) [20]. Compared with the EWT-H zeolite, the position of the characteristic peak of Q^4^ of the samples U-60-08-10/U-60-08-7.3/U-90-08-10/U-90-08-7.3 remains unchanged, and the chemical shift of -103/-93 could be ascribed to different inequivalent T-sites of the zeolite frameworks, which may generate more acid centers (Figure 13a). This is consistent with the acidic results (Table 3).

It suggests that part of boron and aluminum elements can occupy the framework of [B,Al]-EWT by the hydrothermal synthesis method to increase the density of medium and strong acid centers and more Brönsted acid centers than EWT zeolite.

### 2.2. The Catalytic Property of [Bal]-EWT in the Ketalization Reaction of Glycerol with CycloHexanone

The requirement of bio-fuels is continuously growing due to diminishing fossil fuel reserves [21]. Biodiesel appears to be one of the most promising and feasible alternatives among the non-conventional sources of energy. However, the biodiesel production ends up with a huge amount of glycerol [22]. Therefore, it is essential to develop viable methods to utilize the huge quantity of glycerol and to add value to the biodiesel production chain [23,24,25]. The glycerol derivatives, such as acetal, ketal and ester can be used in the cosmetics, plastic, fuel additives and fine chemical industries [25,26,27,28,29]. Cyclohexanone glycerol ketal is a fragrance with flower and woody scent, and has the advantages of an abundant raw material source, simple production process and stable chemical properties. It can be used as a raw material for the synthesis of acrylic cycloheterolipids to prepare light-curing reactive diluents [30]. The traditional catalysts for the ketal reaction are inorganic acids (such as H_2_SO_4_, HCl, H_3_PO_4_, etc.) and L acid. These catalysts will generate a large amount of waste acid, which causes equipment corrosion, environmental pollution, and difficult product separation [31]. Therefore, it is necessary to develop an inexpensive, efficient and reusable solid catalyst.

Zeolites are promising materials as durable and practical heterogeneous catalysts. Mota et al. compared the activities of several zeolites with Amberlyst-15 towards the acetalization of glycerol with formaldehyde [32]. The higher yields were obtained using Hbeta with Si/Al = 16, compared with Amberlyst-15, ZSM-5 and Y zeolites. The poor catalytic activity of the ZSM-5 and Y zeolites can be explained in terms of the narrow pore structure and the hydrophilic character respectively. Dealuminated and desilicated Hbeta zeolites were prepared and applied to the acetalization of glycerol by Venkatesha et al. [33] and Bokade et al. [34], respectively. Both groups concluded that the enhanced catalytic performances were due to the increased pore volumes by dealumination or silication. EWT zeolite has super-large pore structure, can increase the diffusion rate of reactant molecules into the channels, which has the potential to improve the catalytic reaction performance. Herein, the optimal process conditions of the reaction were investigated with H-Beta zeolite [*n*(SiO_2_)/*n*(Al_2_O_3_) = 20] as catalyst, then the catalytic performance of [B,Al]-EWT zeolites were evaluated, and recyclability test of [B,Al]-EWT zeolite was carried out to assess the stability of the material.

**Figure 14:**
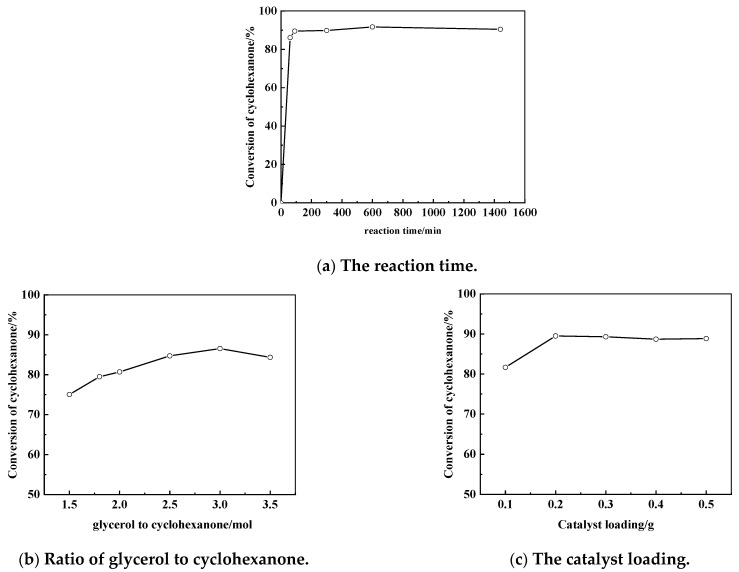

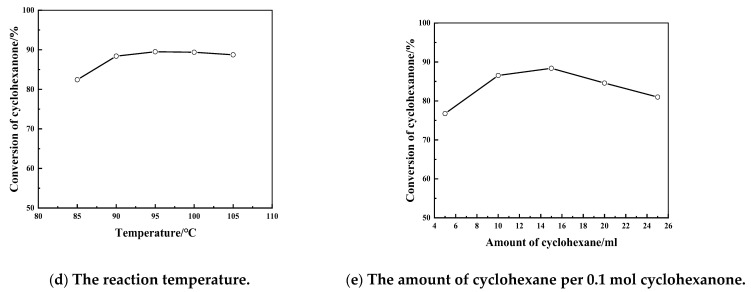
Effects of reaction conditions on conversion of cyclohexanone. The reaction conditions are: (**a**) 0.2 g loading, 95 °C, 15 mL cyclohexane/0.1 mol cyclohexanone, 3.0:1.0 molar ratio of glycerol to cyclohexanone; (**b**) 0.2 g loading, 95 °C, 90 min, 10 mL cyclohexane/0.1 mol cyclohexanone; (**c**) 95 °C, 90 min, 15 mL cyclohexane/0.1 mol cyclohexanone,3.0:1.0 molar ratio of glycerol to cyclohexanone; (**d**) 0.2 g loading, 90 min, 15 mL cyclohexane/0.1 mol cyclohexanone, 3.0:1.0 molar ratio of glycerol to cyclohexanone; (**e**) 0.2 g loading, 95 °C, 90 min, 3.0:1.0 molar ratio of glycerol to cyclohexanone.

In all the cases, formation of two isomeric five-membered and six-membered cyclic ketals were observed, as reported in the literature [35,36]. Using HBeta zeolite as the catalyst, the optimal reaction conditions were determined for the ketal reaction. The cyclohexanone conversion was studied again time at 1:3 molar ratio of glycerol to cyclohexanone, 15 mL cyclohexane per 0.1 mol cyclohexanone and 95 °C, as shown in Figure 14a. It was seen that the catalyst achieved conversion of 86.11% within 90 min of reaction time. Thereafter, with an increase in reaction time, the conversion increases gradually and reaches maximum conversion after 600 min. Upon further extending the reaction time to 1440 min, a decrease in conversion was observed, which may be due to the hydrolysis of cyclohexanone by the water produced during the reaction [37]. The impact of the ratio of glycerol to cyclohexanone was examined at 90 min, leaving the other reaction conditions unchanged, as shown in Figure 14b. It was observed that conversion increases steadily with an increase in the glycerol to cyclohexanone ratio from 1.5:1 to 3.0:1. In the presence of excess glycerol, equilibrium is shifted towards the product side via improving the accessibility to cyclohexanone. However, a slight drop in conversion occurred when the ratio was increased from 3.0:1 to 3.5:1, which may be due to the saturation of active sites by excess cyclohexanone [37]. The effect of catalyst loading on the reaction was studied with 90 min reaction time and glycerol to cyclohexanone ratio of 3.0:1, maintaining other reaction conditions unchanged, as presented in Figure 14c. It was observed that the conversion increased with the increasing catalyst loading till 0.2 g. The improvement may be due to the accessibility of larger amounts of acidic sites in the catalyst for the protonation of cyclohexanone [37]. However, a further increase in the catalyst amount from 0.2 g to 0.5 g results in the conversion dropping a little due to the hydrolysis of the products. Hence, 0.2 g loading was optimal for achieving efficient performance; the effect of temperature in a reversible reaction plays a decisive role in controlling the direction of the reaction. In Figure 14d, it was seen that conversion reached the maximum value (86.11%) at 95 °C but tended to decrease (86.11% to 83.72%) with an increase in temperature from 95 °C to 105 °C. The glycerol ketal reaction is exothermic in nature [38], increasing temperature shifted the equilibrium to the reverse direction, which causes a decrease in glycerol conversion. The results show that the suitable temperature is 90 °C to 100 °C; The water needs to be continuously removed from the reaction system in time. The concentration of the reactant is increased, which is the driving force to speed up the reaction. The solvents, such as cyclohexane, toluene, o-xylene and mesitylene were effective, with cyclohexane being observed to be the most efficient solvent [17]. The cyclohexanone conversion was investigated against the amount of cyclohexane, as shown in Figure 14e. It was observed that conversion increased with an increase amount of cyclohexane per mol cyclohexanone from 5 mL to 15 mL but decrease with the amount of cyclohexane from 15 mL to 25 mL. A. Corma et al. [39] indicate that solvent has not only a positive effect due to more efficient water removal from the reaction, but it has also the benefit of helping to remove the reaction products that adsorbed within the pore of catalysts blocking the access of reactants to active sites. If the amount of solvent is too much, the heat consumption and product loss may increase. The results show that the suitable amount is 10–15 mL. Based on these results, the optimal reaction conditions were determined as follows: 90 min reaction time, 3.0:1.0 molar ratio of glycerol to cyclohexanone, 0.2 g loading, 95 °C to 105 °C, 10–15 mL cyclohexane/0.1 mol cyclohexanone.

After establishing the optimized reaction conditions, the catalytic performances of zeolites were summarized in Table 4 (The detailed calculation of conversion in Table S1). The main observations of the activity study are as follows: (i) The activity order of the catalysts in terms of conversion was Hbeta > HZSM-5 > EWT; (ii) The cyclohexanone conversion rate per mol acid site followed the order EWT > HBeta > HZSM-5, as presented in Table 4, which is consistent with the literature results [32]. EWT zeolite exhibits the highest reaction rate among samples because of the super-large pore channel, which makes the diffusion of reactants and products on the catalytic active site easier. There are more catalytic active sites on the EWT zeolite that can be utilized to catalyze this reaction; (iii) For [B,Al]-EWT zeolites, the activity followed the order U-90-08-10-HCl > U-90-08-10 > U-90-08-7.3 > U-60-08-10 > U-60-08-7.3 >> EWT, as presented in Table 4. Especially, U-90-08-10-HCl zeolite exhibited the highest conversion of cyclohexanone (88.17%) than that of [B,Al]-EWT and HBeta zeolites. This result clear shows an apparent correlation between the increasing trend of activity and the increasing order of the B/L ratio of the catalysts studied, which provides an indication of the vital role of Brönsted acid sites in the ketal reaction; (iv) Irrespective of the zeolites used, the five membered cyclic product (PA) was the major product. The observations indicated the important role of acidic sites and their accessibility in the ketal reaction. Moreover, there was no significant change in the product distribution for different zeolites used.

**Scheme 1:**
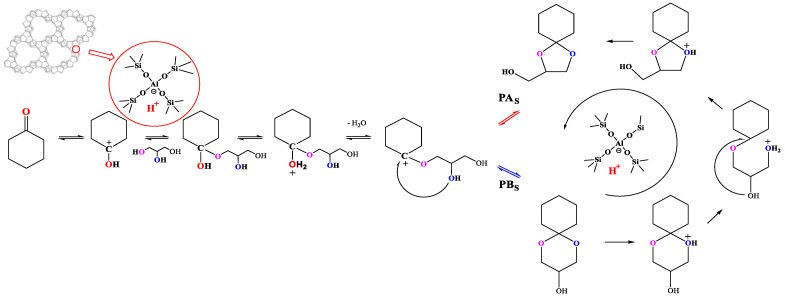
The reaction mechanism of ketal reaction.

Based on the experimental results and previous literature reports [39], the reaction mechanism was proposed in Scheme 1. The ketalization of glycerol over the acid catalysts take place through the initial activation of the cyclohexanone by chemisorption over the Bronsted acid sites. The activated carbonyl carbon then undergoes nucleophilic attack by the primary -OH group of glycerol, forming a hemiketal intermediate. Dehydration of the intermediate leads to the final five- and six-membered ring ketal. Formation of the five-membered ring product involves bond formation between the carbonyl oxygen and the β-carbon of glycerol, accompanied by dehydration. In contrary, formation of the six-membered ring requires bond formation between the carbonyl oxygen and the primary carbon of glycerol. For the cyclohexanone, the five-membered cyclic compound was the major product.

**Figure 15:**
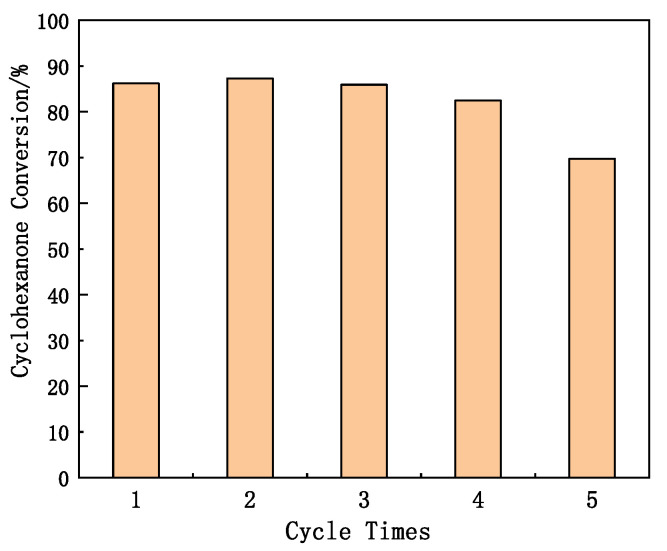
The stability of the U-90-08-10 zeolite of ketalization reaction of glycerol with cyclohexanone.

The stability of the U-90-08-10 zeolite was checked for ketalization of glycerol with cyclohexanone by applying the used catalyst (Figure 15). The study was carried out for five consecutive cycles. In every cycle, the catalyst was tested under optimized reaction conditions. After each run, the used catalyst was separated from reaction mixture and washed with ethanol several times to remove adsorbed species, followed by drying at 373 K for 12 h. Insignificant decrease of conversion was observed up to the fourth cycle, which could be attributed to the small loss of the catalyst during the purification stage. After the fourth run, the activity was decreased to 69.73%. The results indicate that [B,Al]-EWT was stable and reusable without any considerable loss of efficiency up to the fourth run, which is comparable to the activity stability of HBeta zeolite [40].

The higher efficiency observed for [B,Al]-EWT compared with Hbeta, is interpreted by the difference of pore size and acidic sites. The EWT ultra-large pore size (10.8 Å × 4.6 Å) is larger than Hbeta (7.6 Å × 6.7 Å), the number of reactant molecules accessible acid centers increased, and the product PA can more effectively ingress and diffuse within the pores of EWT, resulting in enhanced catalytic performances of [B,Al]-EWT.

## 3. Experiment and Methods

### 3.1. Synthesis of Organic Structure-Directing Agents as Bromide salts

1,1,5,5-tetramethyl-1,6-diazacycloundecane-1, 6-diium bromide salt (TDUB) was prepared by reacting 1,4-dibromobutane (98%, Alfa Aesar in Shanghai, China) with equimolar *N,N,N’,N’*-tetramethyl-1,5-pentanediamine (99%, Acros Organics in Geel, Belgium ) in isopropanol as a solvent with rapid stirring at reflux. The reaction was monitored by HPLC and was completed within about 8 h. The product was precipitated by cooling the reaction mixture to room temperature, adding ethyl acetate. The precipitate was further washed with ethyl acetate/diethyl ether solution. The product was isolated by filtration, and due to the hygroscopic nature, after purification, the diquaternary ammonium salt was stored in a desiccator.

### 3.2. Anion Exchange from Bromide to Hydroxide

The 1,1,5,5-tetramethyl-1,6-diazacycloundecane-1,6-diium bromide salt (38.6 g, 103.2 mmol) was dissolved in water (450 mL) and was passed through an ion-exchange resin (700 mL, corresponding to 840 mmol of exchange capacity) packed in a column. Fractions of pH > 13 were collected and the aqueous solution was concentrated to 90 mL to give 0.102 M (as OH^−^) of 1,1,5,5-tetramethyl-1,5-diazacycloundecane-1,5-diium dihydroxide (TDUH) based on titration of the resulting solution. The yield was 89.4%.

### 3.3. [B,Al]-EWT Zeolite by Direct Synthesis

The synthesis mixture was prepared by combining NaAlO_2_ (149 g∙L^−1^ NaOH, 102 g∙L^−1^ Al_2_O_3_, Laboratory made), silica (40~200 mesh, Qingdao Haihua incorporation, China), template, boric acid (Alfa Aesar Incorporation, Shanghai, China, 99.99%) and deionized water. The final gel composition was 0.15SDA: 0~0.10 NaOH: 1/40~1/70 Al_2_O_3_: 1/30~1/100 B_2_O_3_: SiO_2_: 6.5~15 H_2_O. After being stirred at room temperature for 2 h, the final synthesis gel was transferred to Teflon-lined 45-mL autoclaves and heated at 423~453 K, with rotation (20 rpm) under autogenous pressure, for 5–6 days. The samples were labeled as U-X-Y-Z prepared with TDUH (X-ratio of SiO_2_/B_2_O_3_, Y-ratio of NaOH/SiO_2_, Z-ratio of H_2_O/SiO_2_).

The solid products were recovered by filtration, washed repeatedly with water, and then dried overnight at room temperature. H-EWT was obtained by ion exchange with NH_4_NO_3_ (4 M) and calcination under 823 K. Acid solution treatment sample (U-X-Y-Z-HCl) was prepared with HCl solution (0.01 M) and dried under 373 K.

### 3.4. Characterization

Phase identity and purity of the solid products were checked by powder XRD patterns using a PANalytical diffractometer with Cu KR radiation from Almelo, the Netherlands. Elemental analysis for Si, Al, and Na was carried out by a Type 3013 X-Ray Fluorescence (XRF) Spectrometer from Nihon Riken Electric Co., Ltd. in Tokyo, Japan. The C, H, and N contents of selected samples were analyzed by using a UNICUBE element analyzer in Hanau, Germany. Thermogravimetric analyses (TGA) were performed in air on a SDTQ600 differential thermal analyzer from TA Instruments in Newcastle, DE, USA. Crystal morphology and size were determined by a Japan Hitachi S-4800 type scanning electron microscope (SEM) in Tokyo, Japan. The N_2_ sorption experiments were performed on a Micromeritics ASAP 2010 Static Nitrogen Adsorption Instrument in Georgia, USA. The measurement method is as following, the sample was placed in the processing system, the vacuum was drawn to 1.33 × 10^−2^ Pa at 350 ° C, and maintained for 15 h to purify the sample. At the liquid nitrogen temperature of −196 °C, the adsorption and desorption amounts of nitrogen were measured under different P/P_0_ conditions, the adsorption-desorption isotherm curves were obtained. Then, the two-parameter BET formula was used to calculate the specific surface area, the adsorption volume below the specific pressure P/P_0_ ≈ 0.98 was taken as the pore volume of the sample. ^13^C MAS NMR spectra at a spinning rate of 4.5 kHz were measured on a Varian UNITY INOVA 500 spectrometer at a ^13^C frequency of 125.767 MHz with a *ð*/2 rad pulse length of 5.0 is, a contact time of 1 ms, and a recycle delay of 5 s. Typically, 6000 pulse transients were accumulated. ^29^Si MAS NMR spectra at a spinning rate of 10.0 kHz were measured at a ^29^Si frequency of 99.352 MHz. The spectra were obtained with an acquisition of ca. 1000 pulse transients, which were repeated with a *ð*/2 rad pulse length of 5.0 is and a recycle delay of 30 or 60 s. The ^13^C and ^29^Si chemical shifts are referenced to TMS. ^27^Al MAS NMR spectra at a spinning rate of 11.0 kHz were recorded at a ^27^Al frequency of 130.336 MHz, with a *ð*/20 rad pulse length of 0.5 is, a recycle delay of 1 s, and an acquisition of about 1000 pulse transients. The ^27^Al chemical shifts are referenced to an Al(H_2_O)_6_ ^3+^ solution. The IR spectra in the OH region were measured on a US BIO-RAD FTS3O00-type FT-IR spectrometer using self-supporting zeolite wafers of approximately 15 mg (1.3 cm diameter) from CA, USA. Prior to IR measurements, the zeolite wafers were dehydrated at 723 K under vacuum to a residual pressure of 10^−5^ Torr for 2 h inside a home-built IR cell with CaF_2_ windows. For IR spectroscopy with adsorbed pyridine, the activated self-supporting wafer was contacted with a pyridine-loaded flow of dry He at 373 K for 0.5 h, evacuated (10^−4^ Torr) at the same temperature for 1 h to remove physisorbed pyridine, and then heated at different temperatures. After each desorption step, the concentrations of Brönsted and Lewis acid sites were determined from the intensities of the IR bands around 1550 and 1450 cm^−1^, respectively. NH_3_-TPD was measured with Micromeritics Autochem II 2920.

### 3.5. Catalytic Experiments

For catalytic comparison, zeolite H-ZSM-5 (MFI) with SiO_2_/Al_2_O_3_ = 49 (Produced by SINOPEC CATALYST Corporation, Beijing, China), H-Beta with SiO_2_/Al_2_O_3_ = 20 (Produced by SINOPEC CATALYST Corporation, Beijing, China) and EWT with SiO_2_/Al_2_O_3_ = 92 (Produced by RIPP, Beijing, China) were converted to its proton form by ion exchange with NH_4_NO_3_ (4M) and calcination under 823 K.

The glycerol and cyclohexanone (C) ketal reaction was performed over zeolites in a 250 mL round bottom flask, with vigorous stirring (~1000 rpm). In a typical run, the catalyst, glycerol, cyclohexanone and cyclohexane were placed in the round bottom flask and the reaction mixture was then heated under stirring. Upon completion of the reaction, the catalyst was separated from the reaction mixture by centrifugation. The solvent firstly was distilled under reduced pressure to remove cyclohexane/cyclohexanone, then was extracted by oil ether (60–90 °C) to remove glycerol, and finally was rotary evaporated to obtain the product, which was a colorless viscous liquid.

The qualitative analysis of the products was performed by Agilent gas chromatography (GC) and gas chromatography-mass spectrometry (GC-MS) from CA, USA, respectively. The GC conditions were FID detector, DB-WAX capillary column (30 m × 0.25 mm × 0.25 μm); inlet and detection chamber temperature was 250 °C, the column temperature adopts programmed temperature, the initial temperature is 40 °C, the temperature was kept at 40 °C for 10 min, rise to 70 °C at a rate of 5 °C/min, hold for 5 min, then rise to 180 °C at a rate of 10 °C/min, hold for 25 min; carrier gas was N_2,_ 20 mL/min, gas was H_2_, 45 mL/min, air was 480 mL/min; injection volume was 0.5 μL; split ratio was 20:1. The result of GC analysis of the product was shown in Figure S1. The peaks with retention times of 51.30 min and 54.20 min are the reaction products, which is the mixture of two isomers, 2-hydroxymethyl-1,4-dioxaspiro[4,5]decane and 3-hydroxy-1,5-dioxaspiro[5,5]decane.

The GC-MS conditions were EI ionization source, 70 eV, scanning range m/z = 12–450, scanning time 1 s, and injection volume 0.2 μL. The result of GC-MS analysis of the product was shown in Figure S2. The fragment of m/z = 141 in the PA was not present in the PB. According to the literature report [39], the product of glycerol and cyclohexanone condensation reaction is mainly 1,4-Dioxaspiro[4.5]decane-2-methanol, that is, the substance with a larger peak area in the gas phase.

**Figure 16:**
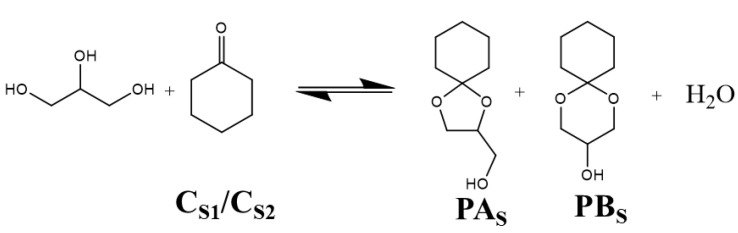
The chemical reaction of ketalization reaction of glycerol with cyclohexanone.

The product was dissolved with ethanol to 10 mL, and the quantitative analysis of the product adopts the standard curve method. The products are 1,4-Dioxaspiro[4.5]decane-2-methanol (PA) and 1,5-Dioxaspiro[5.5]undecan-3-ol (PB), and PA is the target product, as shown in Figure 16.

The mass of *C* in the raw material is recorded as *C_S_*_1_, and the mass of *C* in the product is recorded as *C_S_*_2_. The mass of PA in the product is recorded as *PA_S_*, and the mass of PB in the product is recorded as *PB_S_*. The conversion of cyclohexanone(*C*) and the selectivity of 1,4-Dioxaspiro[4.5]decane-2-methanol (*PA*) are calculated as follows:

(1) $$X_{C}(\%)=\frac{(C_{s1}-C_{s2})g}{(C_{s1})g}\times 100\%$$

(2) $$S_{PA}(\%)=\frac{(PA_{S})g}{(PA_{S}+PB_{S})g}\times 100\%$$

The detailed data of conversion were shown in Table S1 and Figure S3.

## 4. Conclusions

The synthesis, physico-chemical properties, and catalytic properties of [B,Al]-EWT zeolite have been reported. Compared to EWT zeolite, [B,Al]-EWT shows medium and strong acid centers, more Brönsted acid sites, and higher B/L ratio. In the ketalization reaction of glycerol with cyclohexanone, the conversion of the samples (U-90-08-10/U-90-H-HCl) is significantly higher than that of the EWT sample, and approaching or exceeding the Beta zeolite. This work reveals that after the acid regulation, ultra-large pore zeolite [B,Al]-EWT can reach or even exceed the catalytic performance of microporous zeolites, which provides an effective method for the industrial application of ultra-large pore zeolites.

## Figures and Tables

**Table 1:** The feed ratio of samples by direct synthesis.

	Samples	Ratio of Feed					Product	Relative Crystallinity ^a^/%
		*n*(SiO_2_)/ *n*(B_2_O_3_)	*n*(SiO_2_)/ *n*(Al_2_O_3_)	*n*(NaOH)/ *n*(SiO_2_)	*n*(SDA)/ *n*(SiO_2_)	*n*(H_2_O)/ *n*(SiO_2_)		
I	U-Al30	30	30	0.10	0.15	10	LEV	/
	U-Al60	60	30	0.10	0.15	10	LEV	/
II	U-B15	15	60	0.10	0.15	10	EWT + MCM-22	/
	U-B30	30	60	0.10	0.15	10	EWT + MCM-22	/
	U-B60	60	60	0.10	0.15	10	EWT + MCM-22	/
	U-B90	90	60	0.10	0.15	10	EWT + MCM-22	/
	U-B∞	∞	60	0.10	0.15	10	EWT + MCM-22	/
III	U-30-08-10	30	60	0.08	0.15	10.0	EWT + MCM-22	/
	U-30-08-7.3	30	60	0.08	0.15	7.3	EWT + MCM-22	/
	U-30-10-7.3	30	60	0.10	0.15	7.3	EWT + MCM-22 + MEL	/
	U-30-12-10	30	60	0.12	0.15	10.0	EWT + MCM-22 + MEL	/
	U-30-12-7.3	30	60	0.12	0.15	7.3	EWT + MCM-22 + MEL	/
	U-60-10-7.3	60	60	0.10	0.15	7.3	EWT + MCM-22	/
	U-60-12-10	60	60	0.12	0.15	10.0	EWT + MCM-22	/
	U-60-12-7.3	60	60	0.12	0.15	7.3	EWT + MCM-22	/
	U-60-08-10	60	60	0.08	0.15	10	EWT	80.6
	U-60-08-7.3	60	60	0.08	0.15	7.3	EWT	71.8
IV	U-90-08-10	90	60	0.08	0.15	10	EWT	77.5
	U-90-08-7.3	90	60	0.08	0.15	7.3	EWT	71.3
	EWT ^b^	∞	90	0.10	0.15	10	EWT	100

^a^ Relative crystallinity of samples was estimated by comparing the peak area of each zeolite sample at 2θ of 5.0°, 8.1°, 8.9° with that of EWT as a reference. ^b^ EWT is a zeolite composed of silica and alumina element.

**Table 2:** The feed ratio and elemental composition of samples.

Samples	Composition of Zeolites ^a^				Surface Area and Pore Volume ^b^					
	*w*(Na_2_O)/%	*n*(SiO_2_)/ *n*(Al_2_O_3_)	*n*(SiO_2_)/ *n*(B_2_O_3_)	*n*(Al)/ *n*(B)	Surface Area (m^2^ g^−1^)			Pore Volume (cm^3^ g^−^^1^)		
					Total	Micro	Meso	Total	Micro	Meso
U-60-08-10	0.65	42.4	150.7	3.6	536	501	35	0.31	0.23	0.08
U-60-08-7.3	0.65	43.7	132.7	3.0	546	490	56	0.37	0.23	0.14
U-90-08-10	0.95	41.4	180.0	4.4	522	481	42	0.32	0.22	0.10
U-90-08-7.3	0.90	45.4	184.7	4.1	516	463	53	0.35	0.21	0.14
U-90-08-10-HCl	/	65.5	492.3	7.5	519	470	49	0.35	0.22	0.13
EWT	0.62	92.0	/	/	578	556	22	0.26	0.23	0.03

^a^*n*(SiO_2_/Al_2_O_3_) and *n*(SiO_2_/B_2_O_3_) of samples were measured by XRF and ICP-AES. ^b^ Surface area and pore volume of samples were determined by nitrogen physisorption.

**Table 3:** The acidity of samples by NH_3_-TPD and Py-IR.

Sample	Acidity by NH3-TPD ^a^ (μmol g^−1^)			Acidity by Py-IR ^b^-200 °C (μmol g^−1^)			Acidity by Py-IR ^b^-350 °C (μmol g^−1^)			The Fraction of Al (IV) Species ^c^
	Total	Weak (100~290 °C)	Strong (290~600 °C)	Bronsted/(μmol·g^−1^)	Lewis/(μmol·g^−1^)	B/L	Bronsted/(μmol·g^−1^)	Lewis/(μmol·g^−1^)	B/L	
U-60-08-10	762.1	421.0	341.1	119.5	71.7	1.7	44.3	144.9	0.3	6.07
U-60-08-7.3	703.3	420.1	283.2	107.1	115.3	0.9	32.0	111.9	0.3	5.22
U-90-08-10	847.3	437.5	409.8	100.7	134.9	0.7	88.7	159.0	0.6	6.73
U-90-08-7.3	711.6	357.2	354.4	99.4	180.5	0.6	54.0	174.5	0.3	5.04
U-90-08-10-HCl	755.8	417.0	338.8	87.4	41.0	2.1	43.4	59.5	0.7	7.02
EWT-H	626.3	264.7	361.6	31.6	50.8	0.6	10.7	68.0	0.2	4.88

^a^ The quantities of weak and strong acid sites determined by NH_3_-TPD were measured by the amounts of ammonia desorbed at 100–290 and 290–600 °C, respectively; ^b^ The quantities of Brönsted and Lewis acid sites determined by Py-IR were calculated from the Py-IR spectra of zeolites by following the procedures reported by Madeira and co-workers ([17], regulation). ^c^ The fractions of Al(IV) species were calculated from relative peak area of chemical shift 55 ppm in Figure 11b.

**Table 4:** Physico-chemical and catalytic properties of HZSM-5/Beta/EWT zeolites.

Samples	HZSM-5	HBeta	EWT	U-60-08-10	U-60-08-7.3	U-90-08-10	U-90-08-7.3	U-90-08-10-HCl
*n*(SiO_2_)/*n*(Al_2_O_3_)	49	20	92	42	44	41	45	65.5
acidity amount ^a^/mmol NH_3_·g^−1^	0.93	1.38	0.66	0.76	0.70	0.85	0.66	0.76
acidity amount with medium and strong strength ^a^/mmol NH_3_·g^−1^	0.50	0.46	0.36	0.34	0.28	0.41	0.27	0.34
Brönsted acidity (350 °C) ^b^/μmol g^−1^	87	62	11	44	32	89	54	43
B/L (350 °C)	2.8	2.5	0.2	0.3	0.3	0.6	0.3	0.7
cyclohexanone conversion ^c^/%	66.86	86.11	66.35	81.80	81.29	86.21	84.54	88.17
cyclohexanone conversion *10^5^/mol acid site with medium and strong strength	6.69	8.39	9.22	12.04	14.84	9.97	15.24	12.82
Selectivity of PA/%	96.56	98.12	94.91	97.51	99.85	97.63	97.78	98.36

^a^ The quantities of acid sites determined by NH_3_-TPD were measured by the amounts of ammonia desorbed at 100−290 and 290−600 °C, respectively; ^b^ The quantities of Brönsted acid sites determined by Py-IR were calculated from the Py-IR spectra of zeolites. ^c^ Reaction conditions: 0.2 g loading, 95 °C, 90 min,15 mL cyclohexane/0.1 mol cyclohexanone, 3.0:1.0 molar ratio of glycerol to cyclohexanone.

## Data Availability

Not applicable.

## Supplementary Materials

The following supporting information can be downloaded at: https://www.mdpi.com/article/10.3390/molecules27175625/s1, Table S1: The catalytic performance of zeolites by standard curve method; Figure S1: The GC analysis result of product; Figure S2: The GC-MS spectra of products A and B; Figure S3: The standard curve method.

## Sample Availability

Samples of the compounds aren’t available from the authors.
