# Supplementary material for: Synthesis of [B,Al]-EWT-Type Zeolite and Its Catalytic Properties
Source: Molecules. 2022 Aug 31;27(17):5625. doi: 10.3390/molecules27175625 (PMC9458039; doi:10.3390/molecules27175625)
Supplement: Supplementary file 1 [file molecules-27-05625-s001.zip › molecules-1882627-supplementary.pdf]

molecules

MDPI

Supporting Information

Table S1. The catalytic performance of zeolites by standard curve method.

Samples	HZSM-5	HBeta	EWT	U-60-08-10	U-60-08-7.3	U-90-08-10	U-90-08-7.3	U-90-08-10-HCl
$n(SiO_2)/n(Al_2O_3)$	49	20	92	42	44	41	45	65.5
total mass of sample after rotary evaporation/g	57.39	29.831	46.50	30.11	30.23	31.28	29.88	27.65
GC peak area of PA	8096.5	6223.4	10546.7	8547.5	8793.6	5807.3	7090.9	5591.7
cyclohexanone addition amount/g	19.67	19.67	19.67	19.67	19.67	19.67	19.67	19.67
mass of remaining cyclohexanone/g	6.519	2.733	6.619	3.579	3.680	2.713	3.041	2.328
cyclohexanone conversion/%	66.86	86.11	66.35	81.80	81.29	86.21	84.54	88.17
Peak area percentage of PA/%	7.770	8.072	7.180	7.856	8.531	7.462	6.682	7.750
Peak area percentage of PB/%	0.277	0.155	0.385	0.201	0.013	0.181	0.156	0.129
Selectivity of PA/%	96.56	98.12	94.91	97.51	99.85	97.63	97.78	98.36

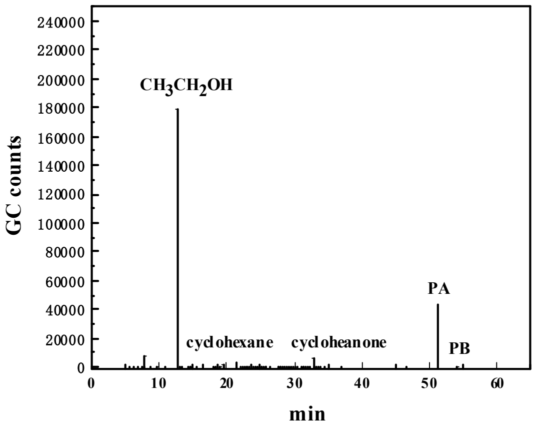

Figure S1. The GC analysis result of product.

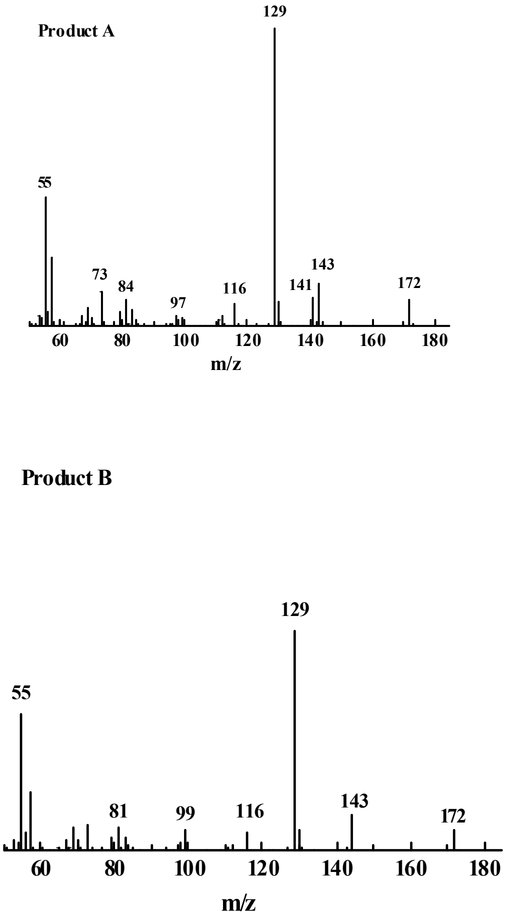

Figure S2. The GC-MS spectra of products A and B.

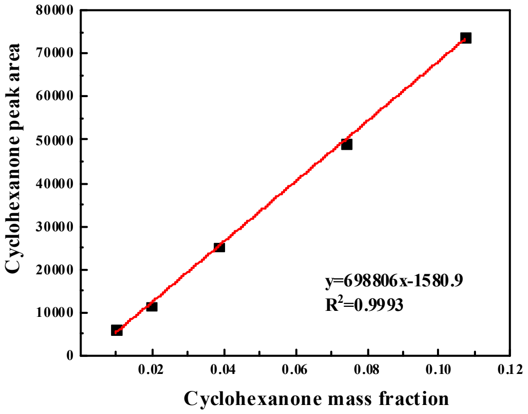

Figure S3. The standard curve method.
